# Are the effects of elevated temperature on meiotic recombination and thermotolerance linked via the axis and synaptonemal complex?

**DOI:** 10.1098/rstb.2016.0470

**Published:** 2017-11-06

**Authors:** Christopher H. Morgan, Huakun Zhang, Kirsten Bomblies

**Affiliations:** John Innes Centre, Colney Lane, Norwich NR4 7UH, UK

**Keywords:** meiosis, recombination, evolution, temperature

## Abstract

Meiosis is unusual among cell divisions in shuffling genetic material by crossovers among homologous chromosomes and partitioning the genome into haploid gametes. Crossovers are critical for chromosome segregation in most eukaryotes, but are also an important factor in evolution, as they generate novel genetic combinations. The molecular mechanisms that underpin meiotic recombination and chromosome segregation are well conserved across kingdoms, but are also sensitive to perturbation by environment, especially temperature. Even subtle shifts in temperature can alter the number and placement of crossovers, while at greater extremes, structural failures can occur in the linear axis and synaptonemal complex structures which are essential for recombination and chromosome segregation. Understanding the effects of temperature on these processes is important for its implications in evolution and breeding, especially in the context of global warming. In this review, we first summarize the process of meiotic recombination and its reliance on axis and synaptonemal complex structures, and then discuss effects of temperature on these processes and structures. We hypothesize that some consistent effects of temperature on recombination and meiotic thermotolerance may commonly be two sides of the same coin, driven by effects of temperature on the folding or interaction of key meiotic proteins.

This article is part of the themed issue ‘Evolutionary causes and consequences of recombination rate variation in sexual organisms’.

## 1. Introduction

Meiosis is a special division during which a cell undergoes two sequential rounds of chromosome segregation with no intervening DNA replication, to generate gamete cells with half the original chromosomal complement. During the first meiotic division (meiosis I), recombination among homologous chromosomes generates novel genetic combinations that play an important role in evolution and breeding. Crossovers persist as cytologically visible chiasmata until homologues segregate in metaphase I and are important for ensuring balanced segregation of homologues [1]. Thus the evolutionarily important effects of recombination and allele shuffling are intimately tied to the physical workings of meiotic chromosome segregation and the maintenance of genome integrity over generations.

Meiotic recombination is an elaborate process, involving numerous steps that take place over the course of many hours (e.g. [2]). Recombination is essentially a DNA repair process that relies on initial programmed double strand break (DSB) formation, followed by repair via numerous recombinases [3]. Nascent recombination events can have several fates, and only a small subset mature as actual crossovers that persist to metaphase as chiasmata [3]. The entire process of meiotic recombination occurs in the context of (and relies on) linear multiprotein structures that form along the chromosomes in meiosis I: the axes, which help unite sister chromatids, and the synaptonemal complex (SC), which subsequently forms between the axes of homologous chromosomes and maintains their tight association as recombination events mature [4]. Perturbations to both structures can affect the fate of the nascent recombination events that are distributed along the chromosomes, and thereby can alter both the final number and position of mature crossover events [5,6].

Meiosis, like many other biological processes, is sensitive to environmental perturbations. Temperature affects the core processes of meiosis which are directly relevant to recombination in at least two important ways: first, temperature is known to alter both the frequency and placement of crossover events, and second, at greater extremes, it can cause disruptions of core structures of the axis and SC that lead to failures in chromosome pairing, synapsis, recombination and segregation [7–9] (reviewed in [10,11]). Some of the effects of temperature are fundamentally similar across species, e.g. temperature can cause shifts in recombination rates and patterns, as well as causing aggregation of axis proteins and failures in synapsis [12]. The failure phenotypes (protein aggregation and mis-assembly into abnormal complexes) seen in meiosis are far from unique to this system. Protein mis-folding and aggregation are well known effects of temperature and occur in a large range of proteins and biological processes [13–15]. The importance of this effect is highlighted by the evolutionary maintenance in virtually all life of conserved extensive ‘proteostasis’ networks that ensure correct protein folding and prevent aggregation [15,16]. Owing to functional constraints, some proteins contain sequences that render them more prone to aggregation than others [14,17]. It may be that the subset of meiosis proteins that forms extended structures (the axis and SC) falls within this class.

Though effects of temperature on meiosis are similar across many eukaryotes and likely constrained by conserved aspects of protein function, the thresholds for meiotic failure as well as the precise temperature ranges that alter recombination vary across species [7]. This makes the important point that even though thermosensitivity is likely an inherent property of at least some of the proteins involved in meiosis, the degree of thermotolerance can evolve [10]. Does the evolution of meiotic thermotolerance occur via modifications of the same proteins and processes that are seen to fail at elevated temperature, or can thermotolerance be altered by modifying regulatory proteins or the expression of e.g. chaperones? While likely multiple factors drive the evolution of meiotic thermotolerance, we support the idea that modification of the structural proteins themselves can have an important role, and that this can link the evolution of thermotolerance with recombination rate variation. Thus we hypothesize that in many cases, variation in recombination rates among species may commonly be a side effect of adaptation of meiosis to the environment.

Understanding how meiotic recombination and axis/SC structure are affected by temperature, and testing the hypothesis that these effects share a common mechanism, is both interesting and timely. Firstly, many studies have indicated that variations in temperature can cause changes in the frequency or position of crossovers, which in turn has implications for evolution as well as breeding [10,11,18]. Secondly, as the optimal temperature range for meiosis is likely to vary between species, presumably in keeping with their native habitats, studying the genetic basis of this adaptation will be important for our understanding of evolution as well as patterns of recombination rate variation [10,19]. Thirdly, identifying how meiosis can be modified to resist temperature extremes could have a positive impact on conservation and global food security in the face of global warming [20]. Furthermore, the question of whether the effects of temperature on meiotic structural stability are related to its effects on recombination rate can shed light on a long-standing debate in evolutionary genetics about why there is variation among populations in recombination rate and whether or not this might be directly adaptive [21].

In this review we first give an overview of some of what is known about the molecular mechanisms that underpin the complex series of events that occur during meiotic recombination, focusing in particular on the structural axes and SC. Then we discuss how these processes and the structures they rely on are affected by temperature. We pay particular attention to the effects of temperature on crossover frequency and the formation of the meiotic axis and SC. Though we discuss evidence from multiple eukaryotes, we focus mostly on plants. We put forward the hypothesis that the effect temperature has on recombination may in many cases be an early symptom of impending meiotic failure at even higher temperature. We also argue that the effect of temperature on recombination may not be a directly adaptive plastic response so much as an unavoidable consequence of the biophysical properties of the proteins that orchestrate key aspects of meiotic recombination. Because of the evidence we discuss below, we favour the idea that variation in recombination rates as measured in the laboratory is in large part a by-product of adaptation to environmental conditions, and not usually itself a selected trait in most systems, though there are a few exceptions, one of the clearest being selection for low recombination in response to within-species genome duplication in plants (reviewed in [10,12]). Finally, because recombination and thermotolerance might often be coupled, we also expect that in those cases in which selection does act directly on recombination rate, thermotolerance may be altered as a pleiotropic effect, and vice versa.

## 2. Mechanisms of meiotic recombination: a brief overview

**Figure 1.**
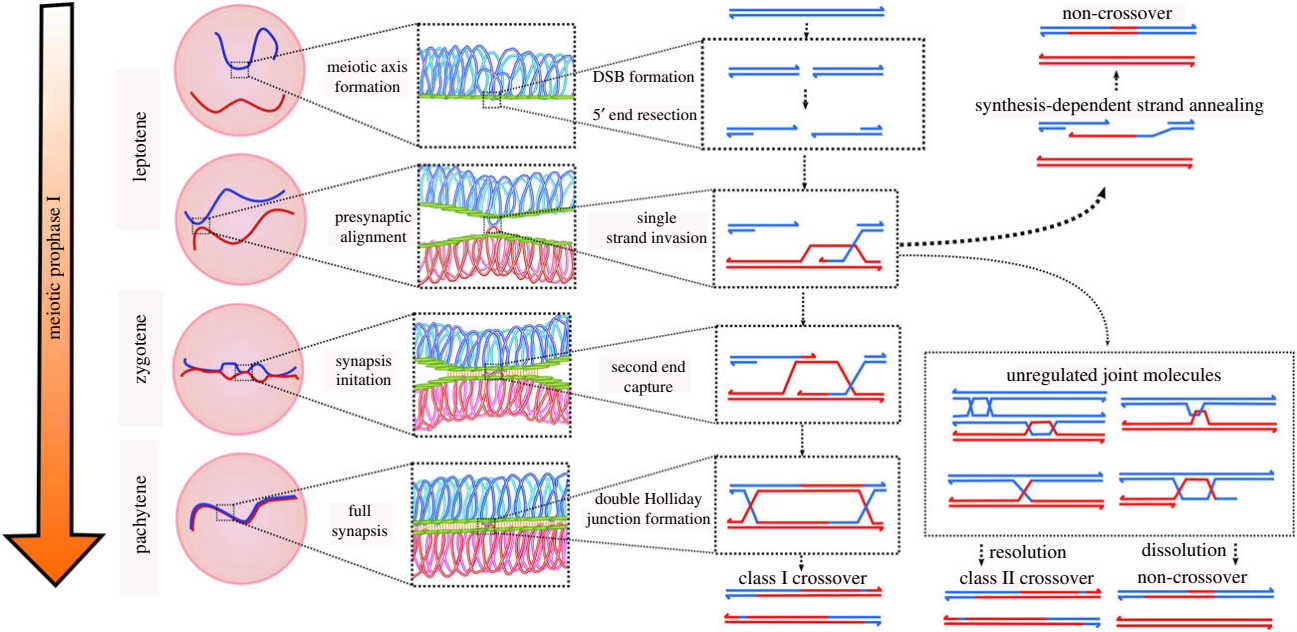
Mechanisms of meiotic recombination. The various stages of recombination that occur during prophase I are illustrated and include global interactions across entire chromosomes as well as more intricate interactions at the single molecule level. Blue and red strands represent homologous chromosomes, and the meiotic axis and synaptonemal complex are coloured green and orange, respectively. The 5′ ends of DNA molecules are represented with an arrowhead.

Meiotic recombination is a carefully orchestrated multi-step process (figure 1) in which many proteins must interact to organize and compact DNA, orchestrate repair, and facilitate chromosome pairing in preparation for even segregation. One of the first steps in recombination is the generation of programmed double-strand breaks (DSBs). Chromosome pairing is dependent on DSB formation, and all that follows is essentially a complex repair process whose outcome is to pair chromosomes and generate some but not too many crossovers among homologues [3]. The molecular mechanisms that underpin initial DSB formation and the subsequent steps of recombination have been studied in a wide variety of model organisms and many elements of recombination are at least at a gross level conserved between species [3]. DSBs are catalysed by the protein Spo11, along with a swathe of additional accessory proteins [22–24]. Following DSB formation, a number of protein complexes are necessary to remove Spo11 and process the ends of breaks to generate long single-stranded DNA tails [25,26]. Once production of the DNA tails is complete, the RecA homologues Rad51 and Dmc1 are loaded onto the ssDNA to form a nucleoprotein filament capable of invading duplex homologous DNA. These long filaments are hypothesized to act as probes that seek out homologous DNA sequences to facilitate chromosome pairing as well as subsequent steps in homologous recombination [27].

After strand-exchange occurs, the majority of recombination intermediates are destined to be resolved as non-crossovers, which are repaired by synthesis-dependent strand annealing or via an alternative recombination dissolution pathway. However, in a smaller number of cases early recombination intermediates progress to form stable crossovers. One way to change crossover numbers could be to regulate the dynamics of this decision point [28]. In most instances, crossover-destined intermediates are resolved via a class I pathway that is dependent upon a group of proteins referred to as the ZMM proteins (Zip3/Zip1/Zip2-Zip4-Spo16, Msh4-Msh5, and Mer3) but occasionally some are also repaired by a class II pathway that is ZMM-independent and, in *Saccharomyces cerevisiae,* requires the nucleases Mus81-Mms4, Yen1 and Slx1-Slx4 [3,29,30]. The process of crossover maturation and resolution requires a large number of proteins working coherently in a highly ordered manner to ensure the process runs in a smooth, error-free manner, and this, presumably, could be sensitive to perturbation by disruption of any of the multiple protein components, and/or protein–protein or protein–DNA interactions.

Critically, the aforementioned recombination steps do not happen in isolation on naked DNA—they occur in the context of, and depend upon, large multiprotein structures that form in prophase of meiosis I called the axis and synaptonemal complex (SC). The proteinaceous axis structure consists of cohesins, condensins and other meiosis-specific proteins. In *S. cerevisiae* the structural backbone of the axis includes Hop1, Red1 and the meiosis-specific cohesin subunit Rec8 [31–33]. In *Arabidopsis thaliana*, there are homologues of the yeast proteins Hop1, Red1 and Rec8 called ASY1, ASY3 and SYN1, respectively [34–36]; homologues are also known from other plant, animal and fungal species (e.g. [37–39]). The axis forms during the leptotene stage of prophase I and tethers replicated sister chromatids into long, looped arrays. In *S. cerevisiae* and other investigated eukaryotes, the meiotic axis plays an integral role in facilitating meiotic recombination. It is especially critical for establishing inter-homologue bias during DNA repair, promoting the use of the homologous chromosome as a repair template rather than the sister chromatid, but is also important for other steps in the maturation of recombination intermediates [40–43]. In yeast, the axis also plays an important role in mediating DSB formation [44], but this role may be less well conserved [35,42].

The axis may also play an additional role in crossover patterning: there is a growing body of evidence suggesting that the meiotic axis may serve as a conduit for transmitting crossover interference [45], which refers to the observation that formation of crossovers is suppressed within regions in close proximity to prior crossovers, causing events to be more widely spaced along chromosomes than would be expected by chance [46]. Evidence supporting a role for the axis in mediating interference includes observations that an axis-associated protein, topoisomerase II, is required for interference in *S. cerevisiae* and that interference requires SUMOylation of the axis protein Red1 [5]. Only the ‘class I’ crossovers mediated by the ZMM proteins are subject to interference; class II crossovers are often referred to as ‘non-interfering’ [45].

During the zygotene stage of prophase I, the axial elements of homologous chromosomes are brought into close juxtaposition via the formation of the SC, a separate proteinaceous structure which acts as a physical linkage along the entire length of the paired homologues [4]. The SC is required for stable homologue pairing and crossover formation [47,48]. In at least some species, the SC may also be important for crossover interference: in *Caenorhabditis elegans*, crossover interference is attenuated when the SC transverse filament protein SYP-1 is partially depleted, and recently the SC was implicated in mediating the dynamic portioning of enzymes involved in meiotic crossover formation [6,49]. However, work in yeast indicates that, at least in this system, interference is set-up prior to full synapsis [50]. It is therefore possible that different species differ in the mechanisms by which interference is established or that both the axis and SC play a role. Importantly, because of their central structural roles, the axis and SC link repair processes of recombination with the physical entities of the chromosomes at large and likely provide an important conduit for the communication required for proper crossover spacing. Mutations that perturb the axis and SC structures thus affect not only chromosome pairing and segregation, but also recombination numbers and patterns (e.g. [48,51]).

## 3. Temperature affects recombination rates and patterns

That there is an effect of varying temperature on crossover frequency and localization has long been recognized (e.g. [52–56]). Various trends of changing crossover frequency in relation to temperature have previously been described [10,57]. The most common trends in studies that investigate a wide range of temperatures appear to be U-shaped curves showing increased recombination rates from some low point as temperature either increases or decreases from normal conditions [58–61]. It is currently unclear whether the increases in recombination at higher and lower temperatures in systems with U-shaped curves have the same underlying cause. Some of the variation in described trends among species may arise from ‘true’ differences, e.g. in which different proteins are most affected by temperature in a given species, but much of it may also arise from variations in experimental design [57], or variation among individuals sampled [62]. Some empirical data from studies investigating the effect of temperature changes on crossover frequency are summarized and reviewed in more detail in [10].

As well as influencing crossover number, it has also been documented that changes in temperature can affect crossover localization. For instance, a study in the spiderwort, *Tradescantia*, indicated that even a subtle increase in temperature can affect crossover distribution [52]. This is also the case in male meiosis in barley, where at 30°C more crossovers are observed in the interstitial regions of the chromosomes than at lower temperatures where they are primarily terminal [55]. Interestingly, at least in barley, the effect of temperature on crossover number and placement is only observed in male meiosis, not female meiosis [63]. Similarly, in an *A. thaliana* mutant for a cyclin-dependent kinase (*CDKG1*, see below) temperature also affects only male meiosis [64]. It may be that differences in the timing or details of recombination between male and female meiosis could lead to differences in thermosensitivity, but what the key differences are remains to be discovered.

The effect of temperature on crossover number and placement is important for interpreting studies that examine only one or a few genetic intervals—movement of crossovers toward or away from chromosome ends could appear as increases or decreases when only single regions are examined, which may also be one of the reasons that many studies that examine just one or a few intervals come to distinct conclusions. That temperature affects recombination in the vast majority of species has an additional interesting implication for interpretation of results: one has to consider what exactly has been measured when two populations are found to differ in recombination rate. If two populations are sampled that have adapted to different temperature regimes, it could be that their temperature response curves are shifted accordingly such that the ‘optimum’ low point falls roughly in the regions at which those populations would normally undertake meiosis. If recombination is then measured in the laboratory we might be sampling populations at different points in their respective response curves, since for at least some of those sampled, the laboratory conditions are a greater deviation from conditions experienced in nature. Recombination rates may thus be higher or lower than they would generally be in natural conditions. The conclusion that wild populations differ in recombination rate based on a laboratory experiment must thus be interpreted with caution. We advocate, therefore, that to really understand how recombination rates differ among populations, particularly of ectotherms, a range of temperatures should be tested.

That temperature is likely an important factor in the evolution of meiosis is further highlighted by the observation that there may be proteins whose role may be specifically to buffer meiosis to the effects of temperature, perhaps acting as some kind of ‘emergency response’ system. Alternatively, these effects could also reflect a generalized decrease in robustness of the system under stress, which could cause mutant effects to become more evident. Several cases of meiotic mutants with temperature-specific effects are known. For example, in *A. thaliana*, mutants for cyclin-dependent kinase G (CDKG1) have fewer crossovers as estimated by counting foci for the MLH1 protein (which marks class I crossovers) as compared with wild-type nuclei at 23°C but not at 12°C, suggesting that CDKG1 might be especially important for maintaining crossover frequency specifically at higher temperatures [64]. In *C. elegans*, three distinct proteins have been shown to have temperature-specific effects on meiosis: IFE-2, PGL-1 and PCH-2 [65–67]. *ife-2* mutants have severely reduced crossover numbers as compared with wild-type worms at 25°C, but not lower temperatures. IFE-2 is thought to stabilize crossover maturation at high temperatures by translationally promoting synthesis of the ZMM proteins MSH-4 and MSH-5 [65]. In the *pgl-1* mutant crossover formation is also compromised at 25°C but not lower temperatures, and this is coupled with a failure of synapsis and aggregation of the SC protein SYP-1, leading to the hypothesis that PGL-1 supports proper SC assembly at higher temperatures either by inhibiting premature meiotic gene expression or by altering levels of heat shock proteins to prevent aggregation [66]. Mutants for *pch-2*, which in its wild-type form is important in chromosome pairing, synapsis and recombination, also show greater defects at higher temperatures [67]. These findings highlight that meiosis can be temperature sensitive in different ways and that either the system becomes more susceptible to the effects of particular mutations under stress, or/and these proteins may have direct roles in mitigating temperature effects.

## 4. Mechanisms of homologous recombination in response to temperature

Why does temperature affect recombination? Some evidence to date hints that the activity of the recombination proteins themselves may in some cases be thermosensitive (discussed below), and that at least in part, the temperature effect may also be due to protein misfolding effects of elevated (or reduced) temperature on the structural axis and SC (discussed in the next section). Finally, there are also hints that other processes such as chromatin structure might also play a role (e.g. [68]), but these are outside the scope of this review.

Temperature may exert effects directly on proteins involved in recombination. For example, in *S. cerevisiae* DSB repair deficiencies in RAD55 or RAD57 mutants are more severe at lower temperatures (23°C) than at 30°C [69]. This indicates that there may be a greater requirement for Rad55/Rad57 mediator function at lower temperatures and that these two proteins could be involved in responding to temperature changes during the strand invasion step of homologous recombination. In mice several meiotic recombinases have strongly reduced activity at both higher and lower temperatures than the normal male meiotic temperature of around 30°C [70]. Class I crossover formation is promoted by the ZMM proteins and it has previously been found that several single and double ZMM mutants in yeast are defective in crossover formation at both high (33°C) and low temperature (23°C), with a severe block in crossover formation at high temperature and slow but efficient crossover formation at low temperature [71]. Another study found that, compared with normal growth conditions of around 28–30°C, the recombination frequency in *mus81* mutants in yeast is slightly increased at 25°C and slightly decreased at 34°C, indicating that the importance of Mus81 in stabilizing class II crossover number also varies with temperature [72]. These studies provide an interesting hint that temperature can alter the function or the importance of particular recombinases. Perhaps species-specific polymorphisms that arise in these genes could lead to different levels of thermotolerance e.g. by modifying expression, protein activity or folding. However, the mechanisms of crossover alteration by temperature in natural systems still remain largely unknown and the relative importance of different meiotic components in the normal response of crossover rates to temperature remains largely unknown.

## 5. Extreme temperature effects on synapsis and meiotic axis formation

Extreme temperatures can exert additional effects on recombination and meiotic stability compared with temperature changes within the tolerated range [57,73]. Across many eukaryotes, a common theme is that failures in synapsis of homologous chromosomes often occur under high temperatures, and in many cases also low temperatures, coupled with sharp declines in recombination [10,53,55,74–76]. One of the most common disruptions observed in a number of species is that components of the SC have been shown to form abnormal structures called polycomplexes at elevated temperatures. For example, Loidl [75] showed that polycomplexes consist of stacked parallel filaments that form interaxial bridges in *Allium ursinum* exposed to 35°C for 60 h. Higgins *et al.* [55] also detected irregularly sized foci of the SC transverse filament protein ZYP1 that were similar to polycomplexes in barley meiocytes at 30°C, but not lower temperatures. High-temperature-induced polycomplex formation is also well described in *C. elegans*: Bilgir *et al.* [66] demonstrated that an increase of only 1.5°C above the standard laboratory incubation temperatures (15–25°C) resulted in the aggregation of SC proteins and concurrent asynapsis. Polycomplexes are thought to occur when successful SC polymerization and synapsis is blocked somehow [49,55,66,75].

**Figure 2.**
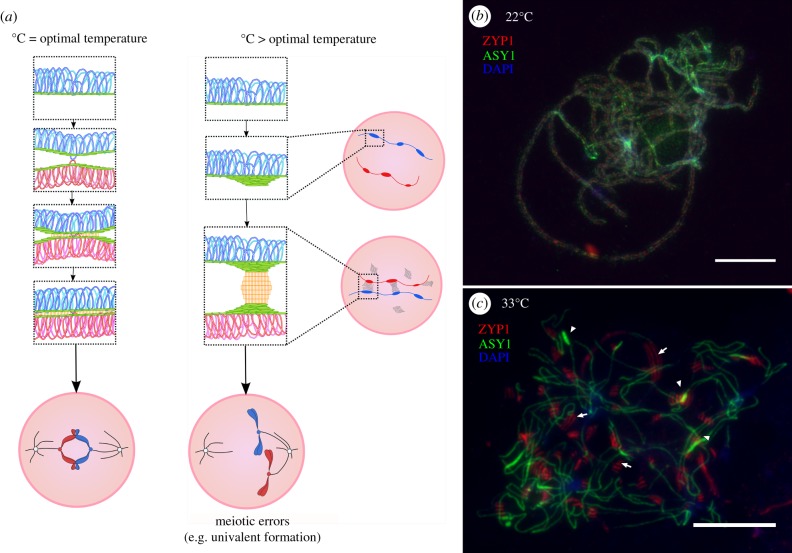
A model for meiotic failure at high temperature. (*a*) Meiotic axis formation and synapsis are compromised at temperatures exceeding the physiological optimum, leading to aggregation of axis proteins and SC polycomplex formation. Perturbation of axis and SC formation leads to downstream effects such as altered crossover frequency and localization or crossover failure leading to univalent formation. (*b*,*c*) Prophase I cells from tetraploid *Arabidopsis arenosa* grown at 22°C (optimal) and 33°C (>optimal) temperatures and stained for the axis-associated protein ASY1 (green), the SC transverse filament protein ZYP1 (red) and DNA (stained with DAPI, blue). (*b*) At 22°C a linear axis is formed and the cell undergoes full synapsis via the formation of a continuous SC. (*c*) At 33°C discontinuous ASY1 staining is observed along with axis-associated ASY1 aggregate formation (white arrowheads) and ZYP1 polycomplexes (white arrows) form in regions that are both linked and unlinked to the axis. Scale bars, 10 µm.

In another case, a recent study by Rog *et al.* [49], investigating the liquidcrystallike properties of the SC, used transmission electron microscopy to show that high-temperature-induced aggregates in *C. elegans* are distinct from those seen in other species or mutants defective in axis organization in that they lack periodic striations and internal order [39]. This suggests that these aggregations result from a temperature-induced denaturation in SC proteins. Whether the denaturation of SC proteins at elevated temperature is unique to *C. elegans* is unclear, but the polycomplexes observed in other species at high temperatures (e.g. figure 2, [75]) do still possess periodic striations and an ordered internal structure, suggesting that denaturation and non-denaturing disruptions of SC organization are distinct abnormalities.

What drives the formation of striated SC polycomplexes at elevated temperatures? SC polycomplex formation may be triggered by, or exacerbated by, prior abnormalities in the axis. Indeed, even at ambient (non-stress) temperatures, abnormalities in the axis can trigger the formation of SC polycomplexes. For example, in *C. elegans*, mutants for any of several axis proteins related to ASY1 and Hop1 show a tendency to form SC polycomplexes [77,78]. This may in at least some cases result from the SC proteins being present in numerical excess relative to their ability to form a fully extended SC: in yeast, for example, absence of DSBs, or of the SC initiation protein Zip3, can also trigger SC polycomplex formation [79]. Even more tellingly, overexpression of the SC central element protein Zip1 by itself can suffice to trigger polycomplex formation [80]. *Caenorhabditis elegans* mutants defective in axis organization can also produce reversible striated SC polycomplexes [49]. Thus if temperature causes defects in the axes, it may be that SC formation is hindered, leading in at least some cases to polycomplex formation. But does temperature cause abnormalities in the axis? Indeed there is evidence that it does. For example, Loidl [75] observed aggregations of meiotic axis material at elevated temperature in *Allium ursinum*, described as ‘thickenings,’ which were often associated with SC polycomplexes. In our own analyses of *Arabidopsis arenosa*, we have observed similar axis thickening at elevated temperatures when examining localization of the axis protein ASY1, confirming that these thickenings are indeed abnormalities of the axis itself (figure 2). As in *Allium*, the axis bulges in *A. arenosa* are often, but not always associated with SC polycomplexes. There is additional evidence for possible axis involvement in temperature effects: a microarray study in barley found that the expression level of an ASY1 homologue is upregulated under high temperature treatment (30°C day; [81]), though whether this plays any role in causing or hindering structural abnormalities of the axis at higher temperatures is not yet known. In *S. cerevisiae*, phosphorylation of one specific serine in Hop1 appears to be required for axis stability only at high temperature [82].

The axis ‘thickenings’ that have been observed at elevated temperature have not been analysed in detail. Likely they are protein aggregations that result from mis-folding or mis-association of one or several axis components. Intriguingly, temperature-associated aggregations of axis proteins are markedly similar to abnormalities observed in both plants and animals that are mutated for the cohesin subunit REC8 (e.g. [83–86]). Cohesins, among other things, are important for the normal emplacement and patterning of the axis though they are not required for the association of axis proteins with DNA [31,87]. Thus it may be that the guiding role of cohesin is important for helping suppress a natural tendency of the axis proteins to self-associate and aggregate. Interestingly, REC8 has been shown to be affected by the oxidative state of the cell, which changes under stress conditions [88], suggesting that cohesin may be a ‘weak link’ in meiosis under stress conditions which in at least some cases could lead to downstream effects on axis and SC structure. In this light it is intriguing that axis and cohesin proteins have been under strong selection in a plant population that adapted to a warmer habitat [19], though it remains to be tested whether the novel alleles in these populations affect thermostability.

High-temperature-induced aggregation of proteins has previously been described in the literature [89]. These abnormalities are not unique to meiosis or even to temperature stress: cellular stress of various types can impair the folding, stability, or interaction of proteins, and can thus affect a range of biological processes and physiological functions (e.g. [13–15]). As proteins of the SC and axis are already prone to self-organizing into large multimeric structures, it may well be that they are more susceptible to aggregation than most proteins. While the cause of the observed abnormalities remains unclear, it could be that disruption of protein interactions that normally promote long linear extension of the axes and SC leaves them to instead self-aggregate into ‘thickenings’ or polycomplexes under stress conditions, particularly when cohesin function is also perturbed.

## 6. Effects of temperature on recombination and axis/synaptonemal complex structure may be linked

Mutations in axis and SC components are known to affect recombination rates and patterns (e.g. [48,51]). Axis and SC structure may be linked to the phenomenon of crossover interference, which can affect crossover placement and number [5,6,45]. While crossover interference is not entirely understood, one model suggests it might be related to the ability of the axis and/or SC to transmit physical tension [49,90]. This in turn would also be something that temperature may influence by altering the dynamics of protein interactions and/or the formation of organized multimeric structures of the axis and SC (e.g. [49]). This hints at the intriguing possibility that as temperatures increase or decrease from some species-specific optimum, the structure of, or interplay between, cohesin and axis components can be altered to first lead to subtle changes in axis emplacement which in turn lead to changes in crossover number and distribution. Changes in axis structure might lead to a change in the strength of interference, or could cause slower crossover designation that could result in a weaker feedback repression of additional DSB and recombination events [91], leading to an increase in crossover number. Ultimately, at greater extremes, the system may no longer be able to buffer the effects of temperature on protein folding, association or aggregation, leading to large-scale structural failures that become cytologically evident. The hypothesis that effects of temperature on recombination might at least sometimes be evolutionarily linked to the thermostability of axis and SC structure is supported by an intriguing observation from grasshoppers: a naturally evolved upward shift in the threshold temperature for meiotic structural failures in a tropical population was associated with suppressed responsiveness of crossover rates to temperature [74,92]. This hints that these traits may indeed be functionally coupled and could sometimes evolve in concert as two sides of the same coin. We hypothesize that meiotic thermotolerance could evolve via structural changes in the axis/SC/cohesin proteins themselves, or by way of their interactions with one another, or through modifications of the proteostasis machinery which helps prevent protein mis-folding and aggregation, and that this would couple recombination rate evolution with the evolution of thermal tolerance thresholds.

## 7. Summary and conclusion

The mechanisms of meiotic recombination are generally well conserved across taxa, even if the proteins involved are often less so. Moreover, though the details may differ, there is a common trend across eukaryotes that temperature can affect recombination rates and positions, as well as cause outright structural failures in meiosis. We hypothesize that in at least some cases these two traits may both result from temperature-induced changes in the structure of the axes and SC, though some variation in temperature trends in recombination could result instead from differences among species in the thermotolerance of recombinases themselves. Though meiosis seems to be affected by temperature in most if not all eukaryotes, often in fairly similar ways, the thresholds for what is a ‘stress’ temperature for meiosis can clearly evolve, though the mechanism remains unknown. Understanding how SC and meiotic axis formation are affected by changing temperatures provides fertile ground for future research and will help in our understanding of how meiosis can be manipulated or can evolve to cope with changing global temperatures and sporadic severe weather events. It will be interesting to examine in detail whether or how the formation of the axis and SC is perturbed as temperatures shift in sub-failure ranges, to determine if our hypothesis that crossover variation and structural failures are functionally linked is correct or not. If these traits are in fact often related, breeding programmes aimed at increasing crossover rates, or shifting crossover placement, will need to take into consideration that by at least some routes, selection for increased recombination may affect thermotolerance in undesirable ways. Moreover, understanding the role temperature adaptation plays in recombination and meiotic evolution will have important implications for considering the nature of, causes, and consequences of recombination rate variation in natural populations.

## Data accessibility

This article has no additional data.
